# Universal eye health: increasing access for the poorest

**Published:** 2013

**Authors:** Elmien Wolvaardt Ellison

**Affiliations:** Editor: Community Eye Health Journal, International Centre for Eye Health, London School of Hygiene and Tropical Medicine, London, UK. **editor@cehjournal.org**

**Figure:**
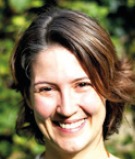
Elmien Wolvaardt Ellison

Eye health is available, but not yet to everyone. The new World Health Organization (WHO) action plan calls for ‘universal eye health’, a new framework to scale up services and expand access to all.

‘Towards Universal Eye Health: A Global Action Plan 2014–2019’ was unanimously adopted by the 194 member states at the World Health Assembly, the decision-making body of the WHO. The International Agency for the Prevention of Blindness (IAPB) has also chosen to highlight universal eye health in its World Sight Day Report 2013.

## What is new in this plan?

The focus of the Global Action Plan is now on ‘universal eye health’: all people should enjoy access to the best quality eye care without the risk that paying for such care will impoverish them.

The plan also sets a specific target of a reduction of 25% in the prevalence of avoidable visual impairment by 2019 (compared to the baseline in 2010).

In principle, universal eye health involves the following:

- offering comprehensive eye care services (for eye health promotion, prevention, treatment, and rehabilitation)
- integrating eye health into the wider health system
- providing access for everyone, including the poor, minorities, and the disabled
- ensuring that payment for services does not prevent access. Services should be free for the poorest – whether by means of fee waivers or national health insurance.

In practice, however, there are many challenges. These include:

- serious shortages in trained personnel, particularly in Africa
- low rates of surgery and irregular outreach to the poorest and rural populations
- treatment costs that are too high for many poor and marginalised people
- barriers, such as transport, lack of appropriate technologies and discrimination. These barriers make it difficult for vulnerable groups of people (the poor, minorities, the disabled, and women) to have access to eye care

**Figure:**
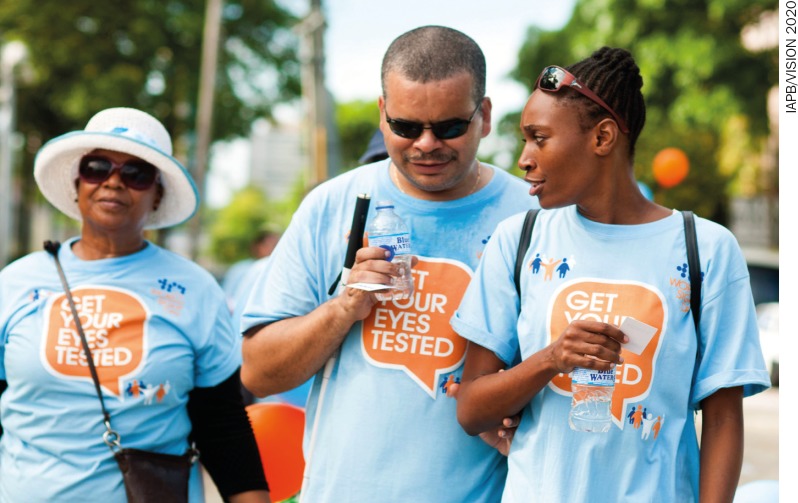
Participants in Trinidad, Tobago ‘walk for sight’ on World Sight Day 2013

## User fees

User fees in public health facilities are a barrier to access and a major obstacle to achieving universal health coverage. To address this barrier, many countries have introduced exemption policies, such as free health care for children or other groups. Other countries have introduced national insurance schemes to help cover the costs of eye care.

## Making progress

In **Africa,** Ghana's National Health Insurance System now covers most ocular diseases, and almost every district has an ophthalmic nurse.^1^ Burkina Faso has made children under five exempt from paying health care fees. As a result, the number of children attending at health facilities has increased six-fold. It is estimated that this could save the lives of around 20,000 children every year, at a cost of just £3 per child per year.^2^

In the **Eastern Mediterranean Region,** Jordan and Saudi Arabia are replacing inflexible hospital-based programmes with comprehensive community-based screening initiatives. Saudi Arabia has incorporated prevention of blindness into its new primary health care policy with a dedicated budget line and training schedule.

In **Europe,** an indicator on eye health was included in England's Public Health Outcomes Framework in 2012 to track the rates of three major causes of avoidable sight loss (glaucoma, macular degeneration and diabetic retinopathy). In **Latin America,** the government of Chile (where 70% of the population is not covered by private insurance) guarantees universal eye health coverage, for example by paying the fees in full if the patient is unable to afford care. In areas where government institutions cannot provide care, private organisations offer services that are paid for by government.

### Source

http://www.iapb.org/news/new-iapb-report-‘universal-eye-health’-launched-world-sight-day

**Time to reflect**

- Has your country adopted the Global Action Plan? It has if it is a WHO member state. Visit **www.who.int/countries/en/** to see if your country is listed.
- What is needed in your district or country in order to provide universal eye health?
- Who are the decision makers who can act to make these changes?
- What can you do to ensure access to eye health care for women, people with physical, sensory, and mental disabilities, people who are poor, people who are unemployed or in the informal sector, and/or refugees?
